# Prediction of donor splice sites using random forest with a new sequence encoding approach

**DOI:** 10.1186/s13040-016-0086-4

**Published:** 2016-01-22

**Authors:** Prabina Kumar Meher, Tanmaya Kumar Sahu, Atmakuri Ramakrishna Rao

**Affiliations:** 1 Division of Statistical Genetics, Indian Agricultural Statistics Research Institute, New Delhi, 110 012 India; 2 Centre for Agricultural Bioinformatics, Indian Agricultural Statistics Research Institute, New Delhi, 110 012 India

**Keywords:** Di-nucleotide association, Machine learning, PWM, Computational feasibility

## Abstract

**Background:**

Detection of splice sites plays a key role for predicting the gene structure and thus development of efficient analytical methods for splice site prediction is vital. This paper presents a novel sequence encoding approach based on the adjacent di-nucleotide dependencies in which the donor splice site motifs are encoded into numeric vectors. The encoded vectors are then used as input in Random Forest (RF), Support Vector Machines (SVM) and Artificial Neural Network (ANN), Bagging, Boosting, Logistic regression, *k*NN and Naïve Bayes classifiers for prediction of donor splice sites.

**Results:**

The performance of the proposed approach is evaluated on the donor splice site sequence data of *Homo sapiens*, collected from Homo Sapiens Splice Sites Dataset (HS3D). The results showed that RF outperformed all the considered classifiers. Besides, RF achieved higher prediction accuracy than the existing methods viz., MEM, MDD, WMM, MM1, NNSplice and SpliceView, while compared using an independent test dataset.

**Conclusion:**

Based on the proposed approach, we have developed an online prediction server (MaLDoSS) to help the biological community in predicting the donor splice sites. The server is made freely available at http://cabgrid.res.in:8080/maldoss. Due to computational feasibility and high prediction accuracy, the proposed approach is believed to help in predicting the eukaryotic gene structure.

**Electronic supplementary material:**

The online version of this article (doi:10.1186/s13040-016-0086-4) contains supplementary material, which is available to authorized users.

## Background

**Fig. 1:**
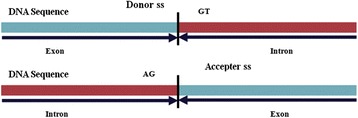
Pictorial representation of donor and acceptor *ss*. Donor *ss* have di-nucleotides GT at the beginning of the intron and acceptor *ss* have di-nucleotides AG at the end of intron

Prediction of gene structures is one of the important tasks in genome sequencing projects, and the prediction of exon-intron boundaries or splice sites (*ss*) is crucial for predicting the structures of genes in eukaryotes. It has been established that accurate prediction of eukaryotic gene structure highly depends upon the ability to accurately identify the *ss*. The *ss* at the exon-intron boundaries are called the donor (5′) *ss* whereas intron-exon boundaries are called the acceptor (3′) *ss*. The donor and acceptor *ss* with consensus GT (at intron-start) and AG (at intron-end) respectively are known as canonical *ss* (GT-AG type; Fig. 1). Approximately, 99 % of the *ss* are canonical GT-AG type *ss* [1]. As GT-and AG-are conserved in donor and acceptor *ss* respectively, every GT and AG in a DNA sequence could be a donor or acceptor *ss*. However, they need to be predicted as either real (true) or pseudo (false) *ss*.

During the last decade, several computational methods have been developed for *ss* detection that can be grouped into several categories viz., probabilistic approaches [2], ANNs [3, 4], SVM [5–7] and information theory [8]. These methods seek the consensus patterns and identify the underlying relationships among nucleotides in *ss* region. ANNs and SVMs learn the complex features of neighborhood nucleotides surrounding the consensus di-nucleotides GT/AG by a complex non-linear transformation, whereas the probabilistic models estimate the position specific probabilities of *ss* by computing the likelihood of candidate signal sequences. Roca et al. [9] identified the di-nucleotide dependencies as one of the main features of donor *ss*. Although the above mentioned methods are complex and computationally intensive, it is evident that position specific signals and nucleotide dependencies are pivotal for *ss* prediction.

**Fig. 2:**
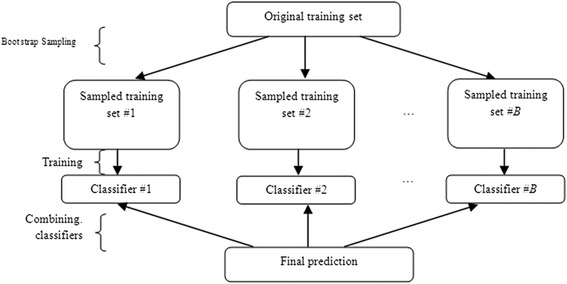
Flow diagram shows the step involved in prediction using ensemble of tree classifiers. Initially, *B* number of samples were drawn from the original training set and a tree was grown using each sample. The final predictions were made by combining all the classifiers

In the class of ensemble classifiers, RF [10] is considered as highly successful one that consists of ensemble of several tree classifiers (Fig. 2). The wide application of RF for prediction purposes in biology can be seen from literature. Hamby and Hirst [11] utilized the RF algorithm for prediction of glycosylation sites and found significant increase in accuracy for the prediction of “Thr” and “Asn” glycosylation sites. Jain et al. [12] assessed the performance of different classifiers (fifteen classifiers from five different categories of pattern recognition algorithms) while trying to solve the protein folding problem. Their experimental results showed that RF achieved better accuracy as compared to the other classifiers. Later on, Dehzangi et al. [13] demonstrated that the RF classifier enhanced the prediction accuracy as well as reduced the time consumption in predicting the protein folds. In the recent past, Khalilia et al. [14] used RF to predict disease risk for eight disease categories and found that the RF outperformed SVM, Bagging and Boosting.

Keeping the above in view, an attempt has been made to develop a computational approach for donor *ss* prediction. The proposed approach involves sequence encoding procedures and application of RF methodology. For given encoding procedures, RF outperformed SVM, ANN in terms of prediction accuracy. Also, RF achieved higher accuracy while compared with existing approaches by using an independent test dataset.

## Methods

### Collection and processing of splice site data

The true and false *ss* sequences of *Homo sapiens* were collected from HS3D [15] (http://www.sci.unisannio.it/docenti/rampone/). The downloaded dataset contains a total of 2796 True donor Splice Sites (TSS) (http://www.sci.unisannio.it/docenti/rampone/EI_true.zip) and 90924 False donor Splice Site (FSS) (http://www.sci.unisannio.it/docenti/rampone/EI_false_1.zip). The sequences are of 140 bp long with conserved GT at 71^st^ and 72^nd^ positions respectively.

**Fig. 3:**
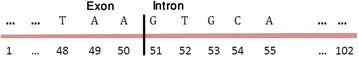
Pictorial representation of *ss* motif. The di-nucleotides GT conserved at 51^st^ and 52^nd^ positions in the *ss* motif of length 102 having 50 nucleotides flanking on both sides of GT

Both introns and exons have important role in the process of pre-mRNA splicing. To be more specific, presence of conserved-ness at both 5′ and 3′ ends of intron as well as exonic splicing enhancers [16, 17] is vital from splicing point of view. Besides, the length of an exon is also an important property for proper splicing [18]. It has been shown in vivo that internal deletion of consecutively recognized internal exons that are below ~50 bp may often lead to exon skipping [19]. As far as the length of an intron is concerned, Zhu et al. [20] carried out the functional analysis of minimal introns ranging between 50-100 bp and found that minimal introns are conserved in terms of both length and sequence. Hence, the window length of 102 bp [50 bp at exon-end + (GT + 50 bp) at intron-start] is considered here (Fig. 3).

Though in longer window length there is a less chance of existence of identical sequences, still we performed redundancy check to remove the identical TSS sequences from the dataset. To train the model efficiently, same number of unique FSS (equal to unique TSS) was considered by drawing at random from 90924 FSS. A sequence similarity search was then performed to analyze the sequence distribution, where each sequences of TSS was compared with the remaining sequences of TSS as well as with all the sequences of FSS and vice versa. The percentage of similarity between any two sequences was computed by assigning a score of 1 and 0 for every match and mismatch in nucleotides respectively, and the same is explained below for two sample sequences.

Sequence 1: ATTCGTCATG

Sequence 2: TCTAGTTACG

Score : 0010110101

Similarity (%)=(5/10)*100=50

Further, we prepared a highly imbalanced dataset consisting of 5%TSS and 95%FSS to assess the performance of RF as well as to compare its performance with that of SVM and ANN.

### Computation of Position Weight Matrix (PWM)

The sequences of both TSS as well as FSS were position-wise aligned separately, using the di-nucleotide GT as the anchor. This position-wise aligned sequence data was then used to compute the frequencies and probabilities of nucleotides at each position. From a given set S of *N* aligned sequences each of length *l*, *s*_*1*_,…, *s*_*N*_, where *s*_*k*_ = *s*_*k1*_,…, *s*_*kl*_ (*s*_*kj*_ є{A, C, G, T}, *j* = 1, 2, …, *l*), the PWM was computed as

1 $$ \begin{array}{l}{p}_{ij}=\frac{1}{n}{\displaystyle \sum_{k=1}^n{I}_i\left({s}_{kj}\right)}\left\{\begin{array}{l}i=A,\;C,\;G,\;T\\ {}\\ {}j=1,2, \dots,\;l\end{array}\right.\\ {}\\ {} where\;{I}_i(q)=\left\{\begin{array}{l}1\kern0.24em  if\;i=q\\ {}\\ {}0\kern0.24em  otherwise\end{array}\right.\;\\ {}\end{array} $$

**Fig. 4:**
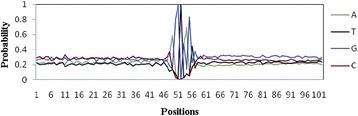
Graphical representation of the PWM for the TSS. The graph shows the probability distribution of four nucleotide bases (ATGC) around the splicing junction

The PWM with four rows (one for each A, C, G, and T) and 102 columns i.e., equal to the length of the sequence (Fig. 4) was then used for computing the di-nucleotide association scores.

### Di-nucleotide association score

The adjacent di-nucleotide association scores are computed under proposed encoding procedures as follows:

1. In the *first* procedure (P-1), the association between any two nucleotides occurring at two adjacent positions was computed as the ratio of the observed frequency to the frequency due to random occurrence of the di-nucleotide. For *N* position-wise aligned sequences, numerator is the number of observed di-nucleotide occurring together, whereas the denominator is *N* times of 0.0625 (=1/16, probability of occurrence of any di-nucleotide at random).
2. In the *second* procedure (P-2), the association was computed as the ratio of the observed frequency to the expected frequency, where expected frequency was computed from PWM under the assumption of independence between the positions.
3. In the *third* procedure (P-3), the di-nucleotide association was computed as the absolute value of the relative deviation of the observed frequency from the expected frequency, where expected frequency was computed as outlined in P-2.

In all the three procedures, the scores were transformed to logarithm scale (base 2) to make them uniform. The computation of the di-nucleotide association scores is explained as follows:

Let *p*_*j*_^*i*^ be the probability of occurrence of *i*^*th*^ nucleotide at *j*^*th*^ position, ${p}_{j^{\prime}}^{i^{\prime }}$ be the probability of occurrence of *i′ *^*th*^ nucleotide at *j′*^*th*^ position and ${n}_{j,{j}^{\prime}}^{i,{i}^{\prime }}$ be the frequency of occurrence of *i*^*th*^ and *i′*^*th*^ nucleotides together at *j*^*th*^ and *j′*^*th*^ positions respectively. Then the different di-nucleotide association scores between *i*^*th*^ and *i′*^*th*^ nucleotides occurring at *j*^*th*^ and *j′*^*th*^ positions under P-1, P-2, P-3 were computed using following formula

2 $$ \begin{array}{l}\left(\mathrm{P}-1\right)\to {s}_{\left(j,{j}^{\prime}\right)}^{\left(i,{i}^{\prime}\right)}={ \log}_2\left(\frac{n_{j,{j}^{\prime}}^{i,{i}^{\prime }}}{N*\;0.0625}\right)\\ {}\left(\mathrm{P}-2\right)\to {s}_{\left(j,{j}^{\prime}\right)}^{\left(i,{i}^{\prime}\right)}={ \log}_2\left(\frac{n_{j,{j}^{\prime}}^{i,{i}^{\prime }}}{N*{p}_j^i*{p}_{j^{\prime}}^{i^{\prime }}}\right)\ \mathrm{and}\\ {}\left(\mathrm{P}-3\right)\to {s}_{\left(j,{j}^{\prime}\right)}^{\left(i,{i}^{\prime}\right)}={ \log}_2\left|\frac{n_{j,{j}^{\prime}}^{i,{i}^{\prime }}-N*{p}_j^i*{p}_{j^{\prime}}^{i^{\prime }}}{N*{p}_j^i*{p}_{j^{\prime}}^{i^{\prime }}}\right|\;\end{array} $$

respectively, where ${s}_{\left(j,{j}^{\prime}\right)}^{\left(i,{i}^{\prime}\right)}$ is the association score, *N* is the total number of sequence motifs in the data set; *i*,*i′*є{A,T, G, C} and *j* = 1, 2, …, (window length-1) and *j′* = *j* + 1. A pseudo count of 0.001 was added to avoid the logarithm of zero in the frequency. For a clear understanding, computation of di-nucleotide association scores is given below, through an example with 5 different sequences.

Positions : 0123456789

Sequence 1: ATAC **GT** CATG

Sequence 2: TGTA **GT** TTCG

Sequence 3: ATGC **GT** ACAC

Sequence 4: GACT **GT** TGCT

Sequence 5: CCTG **GT** GAGA

Using these sequences, the random, observed and expected (under independence) frequencies for di-nucleotide AT occurring at positions 0, 1 respectively are computed as follows:

Observed frequency = Number of times AT occurs together at 0^th^ and 1^st^ positions respectively

=2

Random frequency = Number of sequences × Probability of occurrence of any of the 16 combinations of di-nucleotides at random (=1/16)

=5*0.0625

=0.3125

Expected frequency under independence = Number of sequences × Probability of independent occurrence of A at 0^th^ position × Probability of independent occurrence of T at 1^st^ position

=5*(2/5)*(2/5)

=0.8

In similar way, the frequencies can be calculated for other possible di-nucleotide combinations (AA, AG, AC, TA, …, CC) occurring at all possible adjacent positions. Now, the association scores for three different procedures P-1, P-2 and P-3 can be calculated by using equation (2) as

$$ \mathrm{P}\hbox{-} 1\to {s}_{\left(0,1\right)}^{\left(A,T\right)}={ \log}_2\left(\frac{Observed}{Random}\right)={ \log}_2\left(\frac{2}{0.3125}\right), $$

$$ \mathrm{P}\hbox{-} 2\to {s}_{\left(0,1\right)}^{\left(A,T\right)}={ \log}_2\left(\frac{Observed}{Expected}\right)={ \log}_2\left(\frac{2}{0.8}\right) $$

and

$$ \mathrm{P}\hbox{-} 3\to {s}_{\left(0,1\right)}^{\left(A,T\right)}={ \log}_2\left|\frac{Observed- Expected}{Expected}\right|={ \log}_2\left|\frac{2-0.8}{0.8}\right| $$

### Construction of scoring matrices

**Fig. 5:**
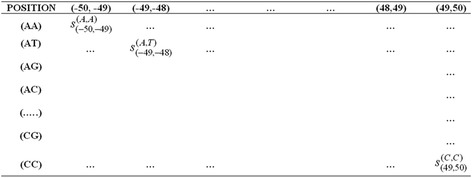
A sample scoring matrix. There are 101 columns for different combination of positions and 16 rows for all possible combinations of nucleotides. This scoring matrix was prepared under all the three encoding procedures

For a sequence of *l*bp long, *l*-*1* combinations of two adjacent positions are possible. Again, in each combination, 16 pairs of nucleotides (AA, AT,…,CG, CC) are possible. Thus, scoring matrices, each of order 16× (*l*-*1*), were constructed using di-nucleotide association scores under all the three procedures. Figure 5 shows a sample scoring matrix for 102 bp window length.

### Ten-fold cross-validation and encoding of splice site sequence

**Fig. 6:**
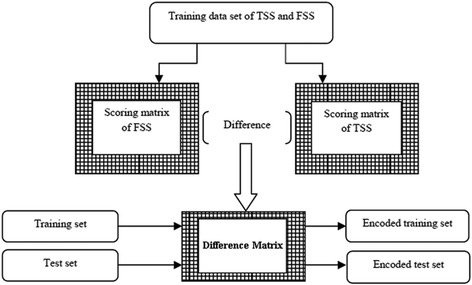
Diagrammatic representation for preparation of encoded training and test datasets from TSS and FSS sequences. For each of the training set in 10 fold cross validation procedure, TSS and FSS scoring matrices were constructed followed by the construction of difference scoring matrices. The encoded training and test sets were obtained after passing the *ss* sequence data of training and test sets through the difference matrix

TSS and FSS sequence datasets were separately divided into 10 random non-overlapping sets for the purpose of 10-fold cross validation. In each fold, one set of TSS and one set of FSS together were used as the test dataset and remaining 9 sets of TSS and 9 sets of FSS together were used as the training dataset. This was performed because 10-fold cross validation procedure is a standard experimental technique for determining how well a classifier performs on a test data set [21]. For each training set, scoring matrices for TSS and FSS were constructed independently and then the difference matrix was derived by subtracting the TSS scoring matrix from the FSS scoring matrix. The training and test datasets were then encoded by passing the corresponding sequences through the difference matrix (Fig. 6), where each sequence was transformed into a vector of scores of length *l*-*1*. A detailed explanation on encoding of the sequence is provided in Additional file 1.

### Classification using Random Forest

**Fig. 7:**
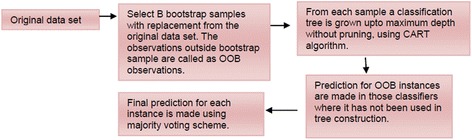
Diagrammatic representation of the steps involved in RF methodology

Let *L*(**y**, **x**) be the learning dataset, where **x** is a matrix of *n* rows (observations) and *p* columns (variables), **y** is the response variable that takes values from *K* classes. Then, the RF consists of ensemble of *B* tree classifiers, where each classifier is constructed upon a bootstrap sample of the learning dataset. Each classifier of RF votes each test instances to one of the pre-defined *K* classes. Finally, each test instance is predicted by the label of winning class. As the individual trees are constructed upon a bootstrap sample, on an average 36.8 % $\left[{\left(1-\frac{1}{n}\right)}^n\approx \frac{1}{e},\left(e\approx 2.718\right)\right]$ of instances do not play any role in the construction of each tree, and are called as Out Of Bag (OOB) instances. These OOB instances are the source of data used in RF for estimating the prediction error (Fig. 7). RF is computationally very efficient and offers high prediction accuracy with less sensitiveness to noisy data. For classification of TSS and FSS, RF was chosen over the other classifiers as it is a non-parametric (i.e., it does not make any assumption about the probability distribution of the dataset) method as well as its ability to handle large data sets. For more details about RF, one can refer [10].

### Tuning of parameters

There are two important parameters in RF viz., number of variables to choose at each node for splitting (*mtry*) and number of trees to grow in the forest (*ntree*). Tuning of these parameters is required to achieve maximum prediction accuracy.

*mtry*

A small value of *mtry* produces less correlated trees that consequently results in lower variance of prediction. Though, integer (log2^(*p*+1)^) number of predictors per node has been recommended by Breiman [10], this mayn’t provide best possible result always. Thus, RF model was executed with different *mtry* values i.e., *1*, √*p*, *20* %*p, *30* %*p, *50* %*p and *p* to find out the optimum one. The parameterization that generated the lowest and stable OOB Error Rate (OOB-ER) was chosen as the optimal *mtry*.

*ntree*

Many times, the number of trees to be grown in the forest for getting the stable OOB-ER is not known. Moreover, OOB-ER is totally dependent on the type of data, where the stronger predictor leads to quicker convergence. Therefore, the RF was grown with different number of trees, and the number of trees after which the error rate got stabilized was considered as the optimal *ntree*.

### Margin function

Margin function is one of the important features of RF that measures the extent to which the average vote for right class exceeds the average vote for any other class. Let (**x**, y) be the training set having *n* number of observations where each vector of attributes (**x**) is labeled with class y_j_ (where, j = 1, 2 for binary class), i.e., the correct class is denoted by y (either y_1_ or y_2_). Further, let *prob* (y_j_) be the probability of class y_j_, then the margin function of the labeled observation (**x**, y) is given by

$$ m\left(\mathbf{x},\ \mathrm{y}\right)= prob\left[h\left(\mathbf{x}\right)=y\right]-\overset{2}{\underset{\begin{array}{l}j=1\\ {}{y}_j\ne y\end{array}}{ \max }} prob\left[h\left(\mathbf{x}\right)={y}_j\right] $$

If *m* (**x**, y) > 0, then *h* (**x**) correctly classifies y, where *h* (**x**) denotes a classifier that predicts the label y for an observation **x**. The value of margin function always lies between-1 to 1.

### Implementation

The RF code was originally written in Fortran by Breiman and Cutler and also included as a package “randomForest” in R [22] and this package was implemented (for execution of RF model) on a windows server (82/GHz and 32 GB memory). Run time was dependent on data size and *mtry*, ranging from 1 second per tree to over 10 seconds per tree.

### Performance metrics

**Fig. 8:**
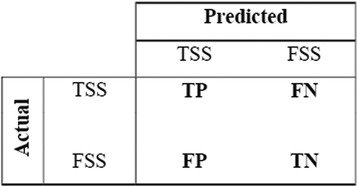
Diagrammatic representation of confusion matrix. TP, FP, TN and FN are the number of true positives, false positives, true negatives and false negatives respectively. TP is the number of TSS being predicted as a TSS and TN is the number of FSS being predicted as FSS. Similarly, FN is the number of TSS being incorrectly predicted as FSS and FP is the number of FSS being incorrectly predicted as TSS

The performance metrics viz., Sensitivity or True Positive Rate (TPR), Specificity or True Negative Rate (TNR), F-measure, Weighted Accuracy (WA), G-mean and Matthew’s Correlation Coefficient (MCC), all of which are the functions of confusion matrix, were used to evaluate the performance of RF. The confusion matrix contains information about the actual and predicted classes. Figure 8 shows the confusion matrix for a binary classifier, where TP is the number of TSS being predicted as TSS and TN is the number of FSS being predicted as FSS, FN is the number of TSS being incorrectly predicted as FSS and FP is the number of FSS being incorrectly predicted as TSS. The different performance metrics are defined as follows:

$$ \mathrm{T}\mathrm{P}\mathrm{R}\ \mathrm{or}\ \mathrm{Sensitivity} = \frac{TP}{TP+FN}\kern0.36em \left( Same\;as\; recall\;for\; binary\; classification\right) $$

$$ \mathrm{T}\mathrm{N}\mathrm{R}\ \mathrm{or}\ \mathrm{Specificity} = \frac{TN}{TN+FP} $$

$$ \mathrm{F}-{\mathrm{measure}}^{\left(\alpha \right)}=\frac{\left(1+\alpha \right)\times recall\times precision}{\left(\alpha \times recall\right)+ precision}\kern0.48em \left(\alpha\;takes\; discrete\; values\right),\ \mathrm{Precision}=\frac{TP}{TP+FP} $$

$$ \mathrm{F}-{\mathrm{measure}}^{\left(\beta \right)}=\frac{\left(1+{\beta}^2\right)\times recall\times precision}{\left({\beta}^2\times recall\right)+ precision}\kern0.24em \left(\beta\;takes\; discrete\; values\right) $$

$$ \mathrm{W}\mathrm{A}=\frac{1}{2}\left(\frac{TP}{TP+FN}+\frac{TN}{TN+FP}\right) $$

$$ \mathrm{G}-\mathrm{Mean} = \sqrt{\left(\frac{TP}{TP+FN}\right)\left(\frac{TN}{TN+FP}\right)} $$

$$ \mathrm{M}\mathrm{C}\mathrm{C}=\frac{\left(TP\times TN\right)-\left(FP\times FN\right)}{\sqrt{\left(TP+FP\right)\left(TP+FN\right)\left(TN+FP\right)\left(TN+FN\right)}} $$

### Comparison of RF with SVM and ANN

The performances of RF was compared with that of SVM [23], ANN [24] using the same dataset that was used to analyze the performance of RF. The “e1071” [25] and “RSNNS” [26] packages of R software were used for implementing the SVM and ANN respectively. The SVM and ANN classifiers were chosen for comparison because these two techniques have been most commonly used for prediction purpose in the field of bioinformatics. In classification, SVM separates the different classes of data by a hyper-plane. In terms of classification performance, the optimal hyper-plane is the one that separates the classes with maximum margin (a clear gap as wide as possible). The sample observations on the margins are called the support vectors that carry all the relevant information for classification [23]. ANNs are non-linear mapping structures based on the function of neural networks in the human brain. They are powerful tools for modeling especially when the underlying relationship is unknown. ANNs can identify and learn correlated patterns between input datasets and corresponding target values. After training, ANNs can be used to predict the outcome of new independent input data [24]. The SVM model was trained with the radial basis function (gamma = 0.01) as kernel. In the ANN model, multilayer perceptron was used with “Randomize_Weights” as initialization function, “Std_Backpropagation” as learning function and “Act_Logistic” as hidden activation function. The 10-fold cross validation was performed for SVM and ANN, similar to RF. All the three techniques were then compared in terms of performance metrics. Also, the MCC values of RF, SVM and ANN were plotted to analyze the consistency over 10 folds of the cross validation. A similar kind of comparison between RF, SVM and ANN was also made using the imbalanced dataset. To handle the imbalanced data, one additional parameter i.e., *cutoff* was used in RF, where 90 % cutoff was assigned to the major class (class having larger number of observations) i.e., FSS and 10 % to the minor class (class having lesser number of observations) i.e., TSS, based on the degree of imbalanced-ness in the dataset. Similarly, one additional parameter i.e., *class.weights* was used in SVM model, and the weights used were 19 and 1 for TSS and FSS respectively (keeping in view the proportion of TSS and FSS in the dataset). However, no parameter to handle imbalanced-ness was found in “RSNNS” package, therefore the same model of ANN was trained using imbalanced data.

In the case of imbalanced test dataset, the performance metrics were computed by assigning weights w_1_ to TP & FN and w_2_ to FP & TN. Here, w_1_ $$ =\raisebox{1ex}{${n}^{FSS}$}\!\left/ \!\raisebox{-1ex}{$\left({n}^{TSS}+{n}^{FSS}\right)$}\right. $$ and w_2_ $$ =\raisebox{1ex}{${n}^{TSS}$}\!\left/ \!\raisebox{-1ex}{$\left({n}^{TSS}+{n}^{FSS}\right)$}\right. $$, where *n*^*TSS*^ is the number of TSS and *n*^*FSS*^ is the number of FSS in the test dataset. Further, the Mann Whitney *U* test at 5 % level of significance was performed to evaluate the difference among the prediction accuracies of RF, SVM and ANN, by using the *stats* package of R-software.

### Comparison with other prediction tools

The performance of the proposed approach was also compared with other splice site prediction tools such as MaxEntScan (http://genes.mit.edu/burgelab/maxent/Xmaxentscan_scoreseq.html), SpliceView (http://bioinfo4.itb.cnr.it/~webgene/wwwspliceview_ex.html) and NNSplice (http://www.fruitfly.org/seq_tools/splice.html) using an independent test set. Besides, three more methods viz., Maximal Dependency Decomposition (MDD), Markov Model of 1^st^ order (MM1) and Weighted Matrix Method (WMM) given under MaxEntScan were also used for comparison. The independent test set was prepared using two different genes (AF102137.1 and M63962.1) downloaded from Genbank (http://www.ncbi.nlm.nih.gov/genbank/) randomly. Comparison among the approaches was made using the values of performance metrics.

### Web server

A web server for the prediction of donor splice sites was developed using HTML and PHP. The developed R-code was executed in the background using PHP script upon the submission of sequences in FASTA format. The web page was designed to facilitate the user for a sequence input, selection of species (human) and encoding procedures. In the server, the model has been trained with human splice site data and the user has to supply only the test sequence (s) of his/her interest to predict the donor splice sites.

## Results

### Analysis of sequence distribution

**Fig. 9:**
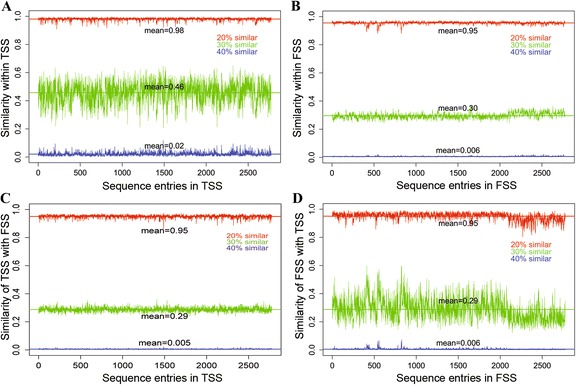
Graphical representation of sequence distribution in the dataset. **a**. Similarities of each sequence of TSS with rest of the sequences in TSS. **b**. Similarities of each sequence of FSS with rest of the sequences in FSS. **c**. Similarities of each sequence of TSS with all the sequences in FSS. **d**. Similarities of each sequence of FSS with all the sequences in TSS. X-axis represents the sequence entries and Y-axis represents fraction of similar sequences

The removal of the identical sequences from the TSS dataset resulted in 2775 unique TSS. A graphical representation of degree of similarity within TSS, within FSS and between TSS & FSS is shown in Fig. 9. It is observed that each sequence of TSS is 40 % (blue) similar with an average of 56 (0.02*2775) sequences of TSS (Fig. 9a) and 15 (0.005*2775) sequences of FSS (Fig. 9c). On the other hand, each sequence of FSS is 40 % (blue) similar with an average of 17 (0.006*2775) sequences of FSS (Fig. 9b) and 17 sequences of TSS (Fig. 9d). Similarly, each sequence of TSS is 30 % (green) similar with an average of 1276 (0.46*2775) sequences of TSS (Fig. 9a) and 805 (0.29*2775) sequences of FSS (Fig. 9c). On the other hand, each sequence of FSS is 30 % (green) similar with an average of 832 (0.30*2775) sequences of FSS (Fig. 9b) and 805 (0.29*2775) sequences of TSS (Fig. 9d). Further, more than 90 % of sequences of entire dataset (both TSS and FSS) are observed to be at least 20 % similar with each other.

### Optimum values of parameters

**Fig. 10:**
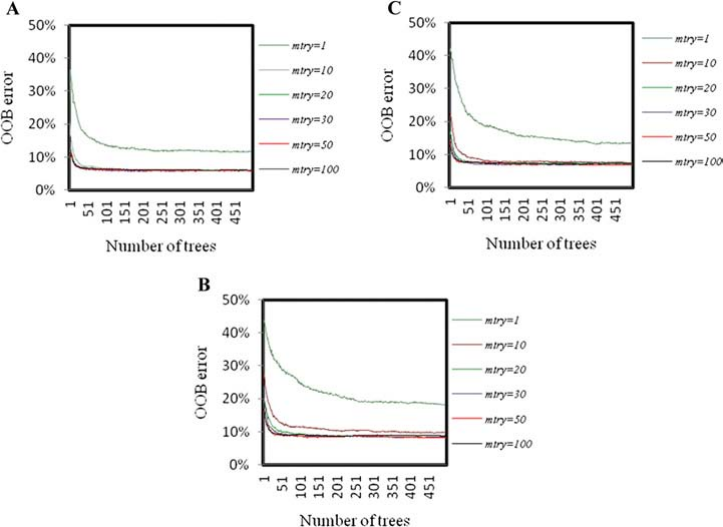
Graphical representation of OOB-ER with different *mtry* and *ntree*. Graphs **a**, **b** and **c** represents the trend of error rates with varying *mtry* for three encoding procedures, P-1, P-2 and P-3. The OOB-ER was minimum for *mtry* = *50* and stabilized with 200 trees (*ntree*)

The graph of OOB error against *ntree* (500) for different *mtry* values is shown in Fig. 10. From Fig. 10 it is observed that the OOB errors are stabilized after 200 trees, for all *mtry* values and that too in all the three encoding procedures. Besides, it is observed that OOB error is minimum at *mtry*=50, irrespective of the encoding procedures. Hence, the optimum values of *mtry* and *ntree* were determined as 50 and 200 respectively. The final prediction was made with optimum values of the parameters.

### Performance analysis of random forest

**Fig. 11:**
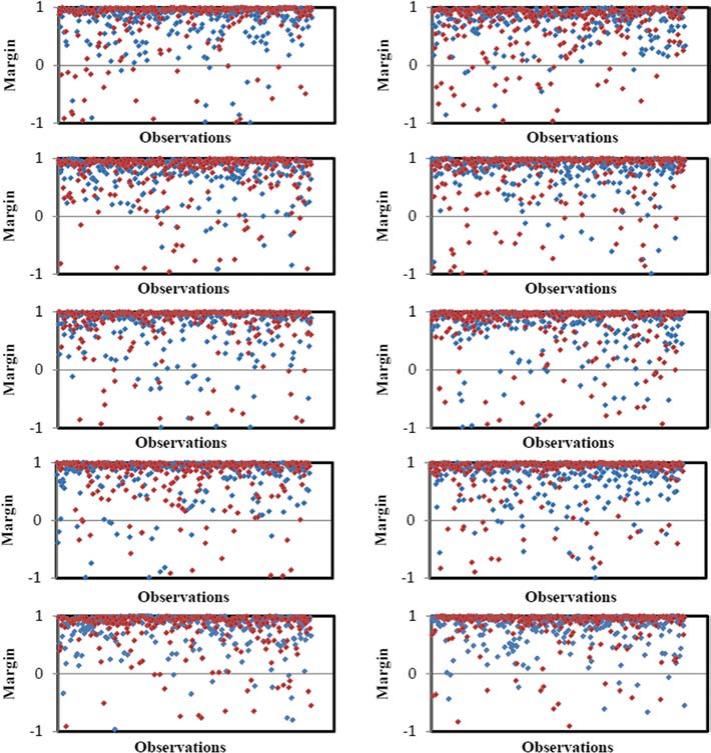
Graphical representation of margin functions for ten-fold cross-validation. Red color points for FSS and blue color for TSS. The instances having value of margin function greater than or equal to zero are correctly predicted test instances and instances having value below zero indicate incorrectly predicted test instances

The plot of margin function for all the 10 folds of the cross-validation under P-1 is shown in Fig. 11. The points in red color in Fig. 11 indicate the predicted FSS and blue color indicate the predicted TSS. The same for P-2 and P-3 are provided in Additional files 2 and 3 respectively. The instances having the values of margin function greater than or equal to zero are correctly predicted test instances and less than zero are incorrectly predicted test instances. From Fig. 11 it is observed that most of the values of margin function are above zero both in TSS and FSS i.e., the RF achieved high prediction accuracy. Similar results are also found in case of P-2 and P-3. Further, the performance of RF is measured in terms of performance metrics and is presented in Table 1. From Table 1 it is seen that the number of correctly predicted TSS is higher than that of FSS, in all the three encoding approaches. Also, it is observed that the average prediction accuracies are ~93 %, ~91 % and ~92 % under P-1, P-2 and P-3 respectively.

**Table 1:** Performance metrics of RF for three encoding procedures

Approaches	Performance Metrics						
	TPR	TNR	F (β = 2)	F (α = 1)	WA	G-mean	MCC
P-1	0.9539	0.9236	0.9313	0.9397	0.9387	0.9386	0.8782
P-2	0.9373	0.9009	0.9108	0.9205	0.9191	0.9189	0.8383
P-3	0.9398	0.9077	0.9163	0.9250	0.9238	0.9236	0.8483

### Comparative analysis among different classifiers

**Fig. 12:**
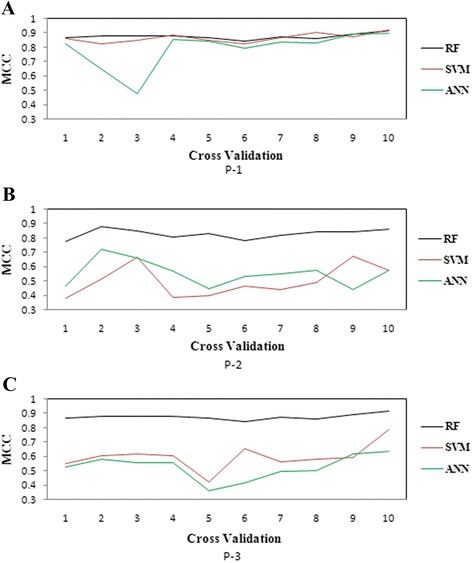
Graphical representation of MCC of the RF, SVM and ANN. MCC is consistent in all the three procedures for the RF over the tenfold cross-validation

The performance metrics of RF, SVM and ANN under P-1, P-2 and P-3 for both balanced and imbalanced training datasets are presented in Table 2. The plots of MCC for RF, SVM and ANN are shown in Fig. 12. From Table 2 it is observed that the prediction accuracies of RF are higher than that of SVM and ANN under both balanced and imbalanced situations. It is further observed that for the balanced training dataset the performances of RF and SVM are not significantly different in P-1 but significantly different in P-2 and P-3 (Table 3). However, the RF performed significantly better than that of ANN in all the three procedures. Furthermore, all the three classifiers achieved higher accuracies in case of balanced training dataset as compared to the imbalanced training dataset. Besides, RF achieved consistent accuracy over the 10 folds under all the three encoding procedures (Fig. 12). On the other hand, SVM and ANN could not achieve consistent accuracies in P-2 and P-3 over different folds of the cross validation.

**Table 2:** Comparison of the performance of RF, SVM and ANN under all encoding procedures with both balanced and imbalanced training dataset

EP	MLA	Balanced Dataset							Imbalanced Dataset						
		TPR	TNR	F (α = 1)	F (β = 2)	G-mean	WA	MCC	TPR	TNR	F (α = 1)	F (β = 2)	G-mean	WA	MCC
P-1	RF	0.954	0.924	0.940	0.932	0.939	0.939	0.878	0.842	0.896	0.865	0.880	0.869	0.869	0.739
		(0.014)	(0.014)	(0.010)	(0.012)	(0.010)	(0.010)	(0.020)	(0.064)	(0.018)	(0.032)	(0.049)	(0.030)	(0.028)	(0.043)
	SVM	0.935	0.930	0.933	0.931	0.933	0.933	0.865	0.104	0.982	0.185	0.349	0.320	0.543	0.180
		(0.015)	(0.017)	(0.015)	(0.015)	(0.016)	(0.016)	(0.031)	(0.027)	(0.018)	(0.041)	(0.031)	(0.040)	(0.013)	(0.061)
	ANN	0.892	0.896	0.894	0.895	0.894	0.894	0.787	0.032	0.988	0.061	0.136	0.178	0.510	0.068
		(0.064)	(0.080)	(0.063)	(0.062)	(0.066)	(0.065)	(0.129)	(0.026)	(0.010)	(0.046)	(0.032)	(0.065)	(0.011)	(0.055)
P-2	RF	0.937	0.901	0.920	0.911	0.919	0.919	0.838	0.883	0.894	0.888	0.891	0.888	0.889	0.777
		(0.020)	(0.016)	(0.016)	(0.018)	(0.016)	(0.016)	(0.033)	(0.038)	()	(0.025)	(0.030)	(0.019)	(0.019)	(0.035)
	SVM	0.720	0.773	0.740	0.752	0.746	0.746	0.493	0.321	0.989	0.482	0.689	0.563	0.655	0.417
		(0.029)	(0.106)	(0.041)	(0.026)	(0.049)	(0.051)	(0.108)	(0.051)	(0.008)	(0.055)	(0.053)	(0.043)	(0.025)	(0.048)
	ANN	0.775	0.777	0.776	0.776	0.776	0.776	0.552	0.305	0.978	0.460	0.661	0.546	0.642	0.383
		(0.067)	(0.037)	(0.049)	(0.059)	(0.048)	(0.045)	(0.090)	(0.049)	(0.014)	(0.052)	(0.051)	(0.043)	(0.022)	(0.046)
P-3	RF	0.940	0.908	0.925	0.917	0.924	0.924	0.848	0.879	0.891	0.884	0.888	0.885	0.885	0.770
		(0.017)	(0.015)	(0.012)	(0.014)	(0.012)	(0.012)	(0.246)	(0.044)	(0.022)	(0.029)	(0.034)	(0.022)	(0.022)	(0.042)
	SVM	0.789	0.807	0.796	0.800	0.798	0.798	0.595	0.249	0.988	0.395	0.609	0.496	0.619	0.352
		(0.044)	(0.068)	(0.042)	(0.042)	(0.046)	(0.045)	(0.090)	(0.052)	(0.008)	(0.062)	(0.056)	(0.049)	(0.026)	(0.055)
	ANN	0.757	0.760	0.758	0.759	0.759	0.759	0.517	0.272	0.979	0.421	0.626	0.516	0.626	0.355
		(0.118)	(0.099)	(0.067)	(0.098)	(0.057)	(0.048)	(0.086)	(0.066)	(0.009)	(0.081)	(0.072)	(0.064)	(0.034)	(0.076)

The values inside the brackets () are the standard errors

*EP* encoding procedure, *MLA* machine learning approaches

**Table 3:** *P*-values of Mann Whitney U statistic for testing the significant difference between RF-SVM, RF-ANN and SVM-ANN at 5 % level of significance for all the performance measures under both balanced and imbalanced training datasets

$D	EP	MLA	TPR	TNR	F (α = 1)	F (β = 2)	G-mean	WA	MCC
Balanced	P-1	RF-SVM	0.02008	0.42473	0.32557	0.04117	0.40550	0.38378	0.32557
		RF-ANN	0.00356	0.73286	0.01854	0.00520	0.02323	0.02569	0.01854
		SVM-ANN	0.01696	0.30585	0.07526	0.03546	0.07526	0.09605	0.10512
	P-2	RF-SVM	0.00018	0.02564	0.00001	0.00001	0.00001	0.00001	0.00001
		RF-ANN	0.00018	0.00018	0.00001	0.00001	0.00001	0.00001	0.00001
		SVM-ANN	0.05869	0.54505	0.16549	0.06301	0.14314	0.14017	0.24745
	P-3	RF-SVM	0.00018	0.00066	0.00001	0.00001	0.00001	0.00001	0.00001
		RF-ANN	0.00018	0.00129	0.00001	0.00001	0.00001	0.00001	0.00001
		SVM-ANN	0.93961	0.16150	0.10512	0.68421	0.07526	0.06954	0.07526
Imbalanced	P-1	RF-SVM	0.00018	0.00018	0.00001	0.00001	0.00001	0.00001	0.00001
		RF-ANN	0.00017	0.00017	0.00001	0.00001	0.00001	0.00001	0.00001
		SVM-ANN	0.00048	0.46778	0.00008	0.00008	0.00008	0.00008	0.00021
	P-2	RF-SVM	0.00018	0.00017	0.00001	0.00001	0.00001	0.00001	0.00001
		RF-ANN	0.00018	0.00018	0.00001	0.00001	0.00001	0.00001	0.00001
		SVM-ANN	0.64854	0.05130	0.39305	0.52885	0.48125	0.32557	0.05243
	P-3	RF-SVM	0.00018	0.00018	0.00001	0.00001	0.00001	0.00001	0.00001
		RF-ANN	0.00018	0.00018	0.00001	0.00001	0.00001	0.00001	0.00001
		SVM-ANN	0.49483	0.05210	0.63053	0.57874	0.57874	0.73936	0.91180

$*D* type of dataset (balanced or imbalanced), *EP* encoding procedures (P-1, P-2, P-3), *MLA* machine learning approaches

Though RF performed better than SVM and ANN, its performance was further compared with that of Bagging [27], Boosting [28], Logistic regression [29], *k*NN [30] and Naïve Bayes [29] classifiers to assess its superiority. The functions *bagging ()*, *ada ()*, *glm ()*, *knn ()* and *NaiveBayes ()* available in R-packages “class” [31], “klaR” [32], “stats” [33], “ada” [34] and “ipred” [35] were used to implement Bagging, Boosting, Logistic regression, *k*NN and Naïve Bayes classifiers respectively. The values of performance metrics, their standard errors and P-values for testing the significance are provided in Table 4, Table 5 and Table 6 respectively. It is observed that the performance of RF is not significantly different from that of Bagging and Boosting in case of balanced dataset (Table 6). On the contrary, RF outperformed both Bagging and Boosting classifiers under imbalanced situation (Table 6). It is also noticed that the classification accuracies (performance metrics) of RF are significantly higher than that of Logistic regression, *k*NN and Naïve Bayes classifiers under both the balanced and imbalanced situations (Table 4, Table 6).

**Table 4:** Performance metrics of Bagging, Boosting, Logistic regression, *k*NN and Naïve Bayes classifiers for all the three encoding procedures under both balanced and imbalanced situations

EP	MD	Balanced							Imbalanced						
		TPR	TNR	F (α = 1)	F (β = 2)	G-mean	WA	MCC	TPR	TNR	F (α = 1)	F (β = 2)	G-mean	WA	MCC
P-1	BG	0.944	0.921	0.934	0.940	0.933	0.933	0.866	0.069	0.996	0.127	0.084	0.258	0.533	0.172
	BS	0.952	0.919	0.936	0.945	0.935	0.935	0.872	0.041	0.898	0.079	0.051	0.192	0.470	0.129
	LG	0.895	0.882	0.889	0.892	0.888	0.888	0.777	0.008	0.993	0.016	0.010	0.087	0.502	0.012
	NB	0.835	0.836	0.836	0.835	0.834	0.835	0.674	0.202	0.838	0.297	0.231	0.409	0.520	0.067
	KN	0.856	0.840	0.847	0.852	0.847	0.848	0.697	0.048	0.854	0.087	0.058	0.200	0.451	0.012
P-2	BG	0.927	0.882	0.907	0.919	0.904	0.904	0.810	0.112	0.992	0.198	0.135	0.330	0.552	0.216
	BS	0.934	0.901	0.918	0.928	0.917	0.917	0.835	0.090	0.996	0.163	0.109	0.296	0.543	0.200
	LG	0.742	0.734	0.739	0.741	0.737	0.738	0.478	0.112	0.981	0.198	0.135	0.330	0.547	0.190
	NB	0.772	0.758	0.767	0.770	0.764	0.765	0.532	0.159	0.884	0.250	0.186	0.373	0.521	0.073
	KN	0.813	0.678	0.760	0.790	0.739	0.746	0.502	0.173	0.981	0.290	0.207	0.412	0.577	0.262
P-3	BG	0.924	0.904	0.915	0.920	0.914	0.914	0.828	0.125	0.991	0.220	0.151	0.351	0.558	0.230
	BS	0.941	0.898	0.922	0.933	0.920	0.920	0.841	0.095	0.995	0.171	0.115	0.305	0.545	0.205
	LG	0.813	0.775	0.798	0.807	0.793	0.794	0.589	0.120	0.983	0.210	0.144	0.342	0.551	0.202
	NB	0.784	0.761	0.775	0.780	0.771	0.772	0.547	0.178	0.945	0.289	0.210	0.410	0.562	0.196
	KN	0.795	0.700	0.756	0.778	0.742	0.747	0.501	0.065	0.989	0.120	0.080	0.247	0.527	0.142

*MD* methods, *EP* encoding procedures, *BG* bagging, *BS* boosting, *LG* logistic regression, *NB* naïve bayes, *KN K* nearest neighbor

**Table 5:** Standard errors of different performance metrics for Bagging, Boosting, Logistic regression, *k*NN and Naïve Bayes classifiers for all the three encoding procedures under both balanced and imbalanced situations

EP	MD	Balanced							Imbalanced						
		TPR	TNR	F (α = 1)	F (β = 2)	G-mean	WA	MCC	TPR	TNR	F (α = 1)	F (β = 2)	G-mean	WA	MCC
P-1	BG	0.0201	0.0178	0.0114	0.0156	0.0113	0.0113	0.0226	0.0234	0.0036	0.0409	0.0282	0.0474	0.0108	0.0334
	BS	0.0146	0.0149	0.0111	0.0125	0.0113	0.0112	0.0224	0.0177	0.3156	0.0334	0.0218	0.0715	0.1652	0.0504
	LG	0.0569	0.0740	0.0601	0.0575	0.0624	0.0621	0.1238	0.0065	0.0056	0.0121	0.0076	0.0313	0.0045	0.0267
	NB	0.0630	0.0826	0.0560	0.0571	0.0573	0.0577	0.1169	0.0357	0.1043	0.0500	0.0390	0.0439	0.0549	0.1579
	KN	0.1502	0.1279	0.1386	0.1454	0.1364	0.1354	0.2701	0.0221	0.3023	0.0389	0.0267	0.0765	0.1595	0.0799
P-2	BG	0.0201	0.0272	0.0192	0.0188	0.0201	0.0200	0.0397	0.0261	0.0060	0.0429	0.0310	0.0421	0.0130	0.0364
	BS	0.0207	0.0179	0.0161	0.0184	0.0163	0.0163	0.0327	0.0273	0.0033	0.0456	0.0325	0.0461	0.0134	0.0358
	LG	0.0688	0.0799	0.0617	0.0644	0.0630	0.0632	0.1272	0.0182	0.0148	0.0290	0.0214	0.0273	0.0107	0.0410
	NB	0.0546	0.0629	0.0421	0.0472	0.0405	0.0407	0.0824	0.0316	0.0733	0.0487	0.0363	0.0436	0.0426	0.1342
	KN	0.0925	0.0811	0.0362	0.0681	0.0266	0.0280	0.0598	0.0235	0.0044	0.0337	0.0269	0.0282	0.0117	0.0270
P-3	BG	0.0156	0.0186	0.0117	0.0130	0.0120	0.0119	0.0237	0.0185	0.0052	0.0291	0.0217	0.0267	0.0089	0.0235
	BS	0.0121	0.0178	0.0102	0.0102	0.0108	0.0107	0.0210	0.0194	0.0039	0.0324	0.0231	0.0323	0.0095	0.0256
	LG	0.0406	0.0586	0.0376	0.0377	0.0409	0.0402	0.0795	0.0210	0.0116	0.0334	0.0247	0.0303	0.0132	0.0440
	NB	0.0380	0.0689	0.0330	0.0323	0.0372	0.0368	0.0735	0.0254	0.0434	0.0397	0.0295	0.0333	0.0286	0.0913
	KN	0.1017	0.0829	0.0629	0.0842	0.0566	0.0544	0.1076	0.0292	0.0078	0.0504	0.0352	0.0629	0.0116	0.0334

*MD* methods, *EP* encoding procedures, *BG* bagging, *BS* boosting, *LG* logistic regression, *NB* naïve bayes, *KN K* nearest neighbor

**Table 6:** *P*-values of the Mann Whitney statistic to test the significant difference between the performance of RF with that of Bagging, Boosting, Logistic regression, *k*NN and Naïve Bayes classifiers in all the three encoding procedures under both balanced and imbalanced situations

$D	EP	CLs	TPR	TNR	F (α = 1)	F (β = 2)	G-mean	WA	MCC
Balanced	P-1	RF-BG	0.343066	0.676435	0.272856	0.212122	0.185711	0.240436	0.272856
		RF-BS	0.820063	0.939006	0.314999	0.795936	0.314999	0.383598	0.314999
		RF-LG	0.001672	0.053092	0.002879	0.000725	0.005196	0.009082	0.005196
		RF-NB	0.000242	0.002796	1.08E-05	1.08E-05	1.08E-05	0.000181	1.08E-05
		RF-KN	0.053182	0.087051	0.028806	0.063013	0.035463	0.025581	0.028806
	P-2	RF-BG	0.41319	0.594314	0.356232	0.356232	0.277512	0.315378	0.356232
		RF-BS	0.837765	0.367844	0.968239	0.968239	0.842105	0.743537	0.842105
		RF-LG	0.000275	0.000439	2.17E-05	2.17E-05	2.17E-05	2.17E-05	2.17E-05
		RF-NB	0.000275	0.004216	2.17E-05	2.17E-05	2.17E-05	2.17E-05	2.17E-05
		RF-KN	0.000376	0.000273	2.17E-05	2.17E-05	2.17E-05	0.000278	2.17E-05
	P-3	RF-BG	0.171672	0.879378	0.14314	0.14314	0.165494	0.15062	0.14314
		RF-BS	0.494174	0.381613	0.970512	0.528849	0.853428	0.820197	0.911797
		RF-LG	0.000181	0.000181	1.08E-05	1.08E-05	1.08E-05	1.08E-05	1.08E-05
		RF-NB	0.000182	0.000279	1.08E-05	1.08E-05	1.08E-05	0.000182	1.08E-05
		RF-KN	0.000182	0.000181	1.08E-05	1.08E-05	1.08E-05	0.000182	1.08E-05
Imbalanced	P-1	RF-BG	0.000269	0.000251	2.17E-05	2.17E-05	2.17E-05	0.000278	2.17E-05
		RF-BS	0.000176	0.002555	0.000181	0.000181	0.000181	0.000178	0.000181
		RF-LG	0.000263	0.000268	2.17E-05	2.17E-05	2.17E-05	0.000263	2.17E-05
		RF-NB	0.000271	0.177338	2.17E-05	2.17E-05	2.17E-05	2.17E-05	2.17E-05
		RF-KN	0.000175	0.025526	1.08E-05	1.08E-05	1.08E-05	0.000182	0.000179
	P-2	RF-BG	0.000179	0.000173	0.000182	0.000182	0.000182	0.000181	0.000182
		RF-BS	0.000181	0.000158	1.08E-05	1.08E-05	1.08E-05	0.000181	1.08E-05
		RF-LG	0.00018	0.000178	1.08E-05	1.08E-05	1.08E-05	0.00018	1.08E-05
		RF-NB	0.000182	0.733634	1.08E-05	1.08E-05	1.08E-05	0.000182	1.08E-05
		RF-KN	0.000181	0.000174	1.08E-05	1.08E-05	1.08E-05	0.000182	1.08E-05
	P-3	RF-BG	0.000176	0.000168	0.000182	0.000182	0.000182	0.000181	0.000182
		RF-BS	0.000179	0.000149	1.08E-05	1.08E-05	1.08E-05	1.08E-05	1.08E-05
		RF-LG	0.000179	0.000177	1.08E-05	1.08E-05	1.08E-05	0.000182	1.08E-05
		RF-NB	0.000177	0.009082	1.08E-05	1.08E-05	1.08E-05	1.08E-05	1.08E-05
		RF-KN	0.00018	0.00018	1.08E-05	1.08E-05	1.08E-05	0.000178	1.08E-05

$*D* data type, *RF* random forest, CLs classifiers, *BG* bagging, *BS* boosting, *LG* logistic regression, *NB* naïve bayes, *KN K* nearest neighbor

### Comparison of RF with other prediction tools

The performance metrics of the proposed approach and the considered existing methods computed by using an independent test dataset is presented in Table 7. It is seen that none of the existing approaches achieved above 90 % TPR. On the other hand, all other approaches (except SpliceView) achieved higher values of TNR than that of proposed approach (Table 7). Furthermore, the proposed approach achieved more than 90 % accuracy in terms of different performance metrics (Table 7).

**Table 7:** The performance metrics for the proposed approach and other published tools using the independent test set

Methods	TPR	TNR	F (α = 1)	F (β = 2)	G-mean	WA	MCC
MaxEntScan	0.627	0.990	0.766	0.884	0.788	0.809	0.662
MDD	0.651	0.991	0.784	0.894	0.803	0.821	0.682
MM1	0.581	0.988	0.730	0.862	0.758	0.785	0.623
WMM	0.415	0.986	0.581	0.764	0.640	0.701	0.488
NNSplice	0.733	0.954	0.824	0.891	0.837	0.844	0.705
SpliceView	0.888	0.879	0.884	0.882	0.883	0.884	0.767
Proposed	0.977	0.922	0.951	0.936	0.949	0.949	0.900

### Online prediction server-MaLDoSS

**Fig. 13:**
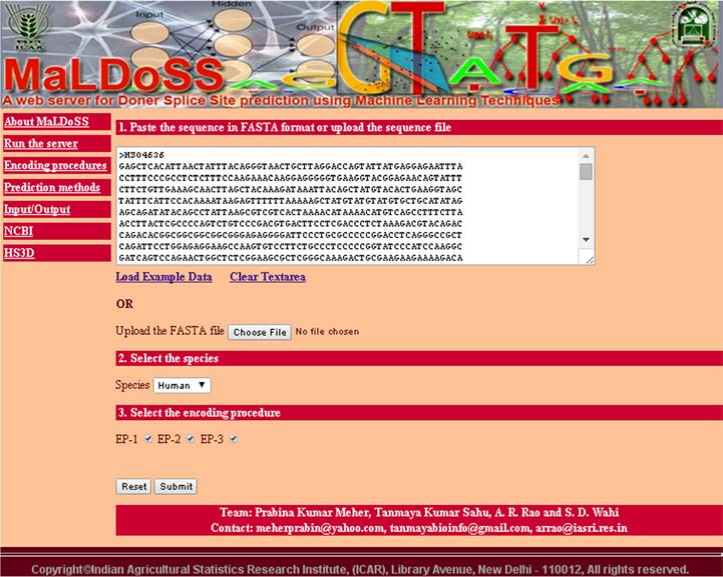
Snapshot of the server page

**Fig. 14:**
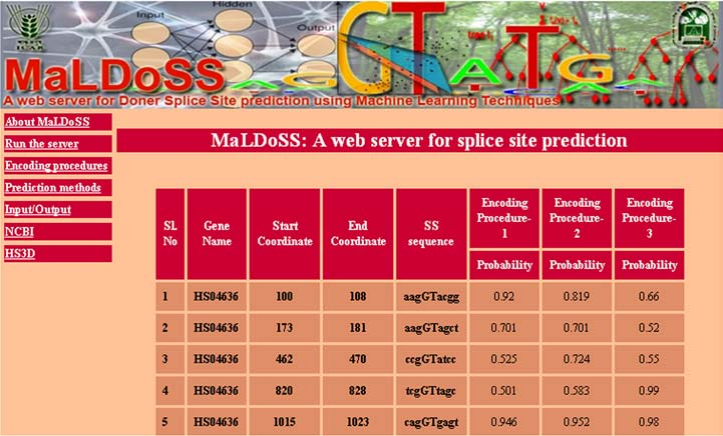
Snapshot of the result page after execution of an example dataset with all the three encoding procedures

The home page of the web server is shown in Fig. 13 and the result page after execution of an example dataset is shown in Fig. 14. Separate help pages are provided as links in the main menu with complete description on encoding procedures and input-output. The gene name, start and end coordinates of splice sites, splice site sequences and probability of each splice site being predicted as TSS are given in the result page. Since RF is observed to be superior over the other classifiers, it is only included in the server for prediction. The prediction server is freely available at http://cabgrid.res.in:8080/maldoss.

## Discussion

Many statistical methods like, Back Propagation Neural Networks (BPNN), Markov Model, SVM etc. have been used for prediction of *ss* in the past. Rajapakse and CaH [4] introduced a complex *ss* prediction system (combination of 2^nd^ order Markov model and BPNN) that achieved higher prediction accuracy than that of Genesplicer [36], but at the same time it is required longer sequence motifs to train the model. Moreover, BPNN is computationally expensive and may increase further with the inclusion of 2^nd^ order Markov model. Baten et al. [6] reported improved prediction accuracy by using SVM with Salzberg kernel [37], where the empirical estimates of conditional positional probabilities of the nucleotides around the splicing junctions are used as input in SVM. Sonnenburg et al. [7] employed *weighted degree* kernel method in SVM for the genome-wide recognition of *ss*, which is based on complex nonlinear transformation. In the present study we applied RF as it is computationally feasible and user friendly. Furthermore, the fine tuning of parameters of RF helps in improving the prediction accuracy.

Most of the existing methods capture position specific signals as well as nucleotide dependencies for the prediction of *ss*. In particular, Roca et al. [9] explained the pivotal role played by the nucleotide dependencies for the prediction of donor *ss*. Therefore, the proposed encoding procedures are based on di-nucleotide dependencies. Further, the earlier *ss* prediction methods such as Weighted Matrix Method (WMM) [38], Weighted Array Model (WAM) [39] and Maximal Dependency Decomposition (MDD) [40] only considered the TSS but not the FSS to train the prediction model. However, FSS are also necessary [41], and hence RF was trained with both TSS and FSS datasets.

There is a chance of occurrence of same *ss* motifs in both TSS and FSS when the length of *ss* motif is small. To avoid such ambiguity, instead of 9 bp long motif (3 from exons and 6 from introns) [42], the longer *ss* motif (102 bp long) was considered in this study. Further, duplicate sequences were removed and a similarity search was performed to analyze the sequence distribution. It is found that each sequence of TSS is 40 % similar with an average of 0.5 % sequences of FSS (Fig. 9c) and each sequence of FSS is 40 % similar with an average of 0.6 % sequences of TSS (Fig. 9d). Also, the sequences are found to be similar (20 % similarity) within the classes (Fig. 9a-b). This implies that the presence of within class dissimilarities and between class similarities in the dataset. Thus the performance of the proposed approach is not over estimated.

The procedure followed in the present study includes WMM and WAM procedures to some extent in finding the weights for the first order dependencies. Besides, the difference matrix captured the difference in the variability pattern existing among the adjacent di-nucleotides in the TSS and FSS. Li et al. [43] have also used di-nucleotide frequency difference as one of the positional feature in prediction of *ss*.

The optimum value of *mtry* was observed as 50, determined on the basis of lowest and stable OOB-ER. This may be due to the fact that each position was represented twice (except the 1^st^ and 102^nd^ positions) in the set of 101 variables (1_2, 2_3, 3_4, …, 100_101, 101_102). Further, OOB-ER was found to be stabilized with small number of trees (*ntree* = 200) and this may be due to the existence of di-nucleotide dependencies in the *ss* motifs that leads to the high correlation between trees grown in the forest. However, we considered the *ntree* equal to 1000 as (i) the computational time was not much higher than that required for *ntree* = *200*, and (ii) the prediction accuracy may increase with increase in the number of trees. Hence, the final RF model was executed with *mtry* = 50 and *ntree* = 1000. The classification accuracy of RF model was measured in terms of margin function, over 10 folds of cross-validation. It is found that the probability of instances being predicted as the correct class over the wrong class is very high (Fig. 11), which is a strong indication that the proposed approach with RF classifier is well defined and capable of capturing the variability pattern in the dataset.

As far as the encoding procedures are concerned, it is analyzed that the dependencies between the adjacent nucleotide positions in the *ss* positively influenced the prediction accuracy. Out of the three procedures (P-1, P-2 and P-3), P-1 is found to be superior with respect to different performance metrics. Though the accuracy of P-2 is observed to be lower than that of P-3, the difference is negligible. Therefore, it is inferred that the ratio of the observed frequency to the random frequency of di-nucleotide is an important feature for discriminating TSS from FSS.

Among the classifiers, RF achieved above 91 % accuracy in all the three encoding procedures, while SVM showed a similar trend only for P-1 and ANN could not achieve above 90 % under any of the encoding procedures (Table 2). The MCC values of RF, SVM and ANN also supported the above finding. Though SVM and ANN performed well in P-1, their consistencies were relatively low in P-2 and P-3 over 10 folds of cross validation. On the other hand, RF was found to be more consistent in all the three encoding procedures. Further, the prediction accuracy of RF was not significantly different (*P*-value > 0.05) from that of SVM, whereas it was significantly higher (P-value < 0.05) than that of ANN in balanced training set under P-1. However, under P-2 and P-3, RF performed significantly better than that of SVM and ANN in both balanced and imbalanced situations (Table 3). Further, the performance of SVM was not significantly different than that of ANN in P-1, whereas it was significantly different in P-2 and P-3 under both balanced and imbalanced datasets (Table 3). In case of imbalanced dataset, RF performed better than SVM and ANN in terms of sensitivity and overall accuracy (Table 2). Besides, the performances of SVM and ANN were biased towards the major class (FSS) whereas RF performed in an unbiased way. Furthermore, all the classifiers performed better under P-2 and P-3 as compared to P-1, in case of imbalanced dataset (Table 2).

Besides SVM and ANN, the performance of Bagging, Boosting, Logistic regression, *k*NN and Naïve Bayes classifiers were also compared with that of RF. Though the performance of RF was found at par with that of Bagging and Boosting in balanced situation, it was significantly higher than that of Logistic regression, *k*NN and Naïve Bayes classifiers. However, in case of imbalanced dataset, RF performed significantly better than Bagging, Boosting, Logistic regression, *k*NN and Naïve Bayes classifiers in all the three encoding procedures. Thus, RF can be considered as a better classifier over the others.

RF achieved highest prediction accuracy under P-1 as compared to the other combinations of encoding procedures (P-2, P-3) and classifiers (SVM, ANN, Bagging, Boosting, Logistic regression, *k*NN and Naïve Bayes). Therefore, the performance of RF under P-1 was compared with different existing tools i.e., MaxEntScan (Maximim Entropy Model, MDD, MM, WMM), SpliceView and NNSplice using an independent test set. The overall accuracy of the proposed approach (RF with P-1) was found better than that of other considered (existing) tools.

The purpose of developing the web server is to facilitate easy prediction of donor splice sites by the users working in the area of genome annotations. The developed web server provides flexibility to the users for selecting the encoding procedures and the machine learning classifiers. As the test sequences belong to two different classes, the instances with probability >0.5 are expected to be true splice sites. Besides, higher the probability more is the strength of instance being a donor splice site. Though, the RF achieved higher accuracy under P-1 as compared to the other combinations, all combinations are provided in the server for the purpose of comparative analysis by the user. To our limited knowledge, for the first time, we have used RF in *ss* prediction.

## Conclusion

This paper presents a novel approach for donor splice site prediction that involves three splice site encoding procedures and application of RF methodology. The proposed approach discriminated the TSS from FSS with higher accuracy. Also, the RF outperformed SVM, ANN, Bagging, Boosting, Logistic regression, *k*NN and Naïve Bayes classifiers in terms of prediction accuracy. Further, RF with the proposed encoding procedures showed high prediction accuracy both in balanced and imbalanced situations. Being a supplement to the commonly used *ss* prediction methods, the proposed approach is believed to contribute to the prediction of eukaryotic gene structure. The web server will help the user for easy prediction of donor *ss*.

### Availability and requirement

MaLDoSS, the donor splice site prediction server, is freely accessible to the non-profit and academic biological community for research purposes at http://cabgrid.res.in:8080/maldoss.

## Additional files

Additional file 1: **An example of the proposed sequence encoding approach.** Description of the data: A precise description about the sequence encoding procedure is provided with an example. (PDF 72 kb)

Additional file 2: **Plotting of margin function for encoding procedure 2 (P-2).** Description of the data: Each dot in the plot is the value of margin function for an observation (TSS or FSS). Ten different plots corresponding to 10 test sets of the 10-fold cross validation. Red and blue points are the values of margin function for FSS and TSS. The values above zero indicate that the instances are correctly classified. (PDF 110 kb)

Additional file 3: **Plotting of margin function for encoding procedure 3 (P-3).** Description of the data: Each dot in the plot is the value of margin function for an observation (TSS or FSS). Ten different plots corresponding 10 test sets of the 10-fold cross validation. Red and blue points are the values of margin function for FSS and TSS. The values above zero indicate that the instances are correctly classified. (PDF 108 kb)

## Abbreviations

ANN: artificial neural network
BPNN: back propagation neural network
EP: encoding procedure
FN: false negative
FSS: false splice sites
HS3D: homo sapiens splice dataset
MCC: matthew’s correlation coefficient
MDD: maximal dependency decomposition
MEM: maximum entropy modeling
MLA: machine learning approaches
MM1: markov model of 1^st^ order
MM2: markov model of 2^nd^ order
OOB: out of bag
OOB-ER: out of bag-error rate
PWM: position weight matrix
RF: random forest
SVM: support vector machine
TNR: true negative rate
TP: true positive
TPR: true positive rate
TSS: true splice sites
WA: weighted accuracy
WAM: weighted array model
WMM: weighted matrix model
