# Supplementary material for: Prediction of donor splice sites using random forest with a new sequence encoding approach
Source: BioData Min. 2016 Jan 22;9:4. doi: 10.1186/s13040-016-0086-4 (PMC4724119; doi:10.1186/s13040-016-0086-4)
Supplement: Supplementary file 1 — An example of the proposed sequence encoding approach. Description of the data: A precise description about the sequence encoding procedure is provided with an example. (PDF 72 kb) [file 13040_2016_86_MOESM1_ESM.pdf]

An example of proposed sequence encoding approach

Initially, a matrix was generated using position-wise aligned true splice site sequence dataset, where the value in each cell corresponds to the score $s_{j,j+1}^{i,i'}$ that represents the dependency between nucleotides *i* and *i'* occurring in the positions *j* and *j+1* (*j*=1, 2, …, L-1). Since length of sequence was 102bp and 16 combinations of di-nucleotides (*i, i'*) are possible, a matrix (having dependency scores) of order 16×101 was generated. Similar another matrix (having dependency scores) of order 16×101 was generated using position-wise aligned false splice site sequence dataset. Then, a difference matrix of order 16×101 was generated by subtracting the dependency matrix of true sites from the dependency matrix of false sites,. The table below represents a hypothetical difference matrix of order 16×101, which is written in transpose form for convenience.

	AA	AT	AG	AC	TA	TT	TG	TC	GA	GT	GG	GC	CA	CT	CG	CC
1 2	0.37	0.34	-0.08	-0.17	1.10	0.64	-0.08	-0.35	-0.13	0.61	0.18	-0.39	-0.02	0.05	-0.86	-0.60
2 3	0.54	0.20	0.05	-0.07	0.79	0.96	-0.12	0.34	-0.50	0.42	0.02	-0.08	-0.30	0.04	-1.32	-0.61
3 4	0.34	0.27	-0.36	-0.26	1.47	0.86	0.36	-0.48	-0.29	0.33	0.01	-0.54	0.03	0.01	-0.62	-0.24
4 5	0.62	0.38	-0.08	-0.01	0.85	0.89	-0.03	0.05	-0.01	0.01	0.27	-0.50	-0.05	-0.39	-0.52	-0.60
5 6	0.41	0.56	0.14	-0.06	0.71	0.77	-0.55	0.35	-0.40	0.27	0.10	0.02	-0.18	-0.18	-1.28	-0.21
6 7	-0.15	0.00	0.15	0.02	0.96	0.78	0.10	-0.21	-0.53	0.36	-0.40	-0.28	0.21	-0.09	-0.90	0.20
7 8	0.52	0.19	-0.30	-0.29	1.35	0.93	-0.43	0.12	-0.35	0.27	-0.18	-0.30	0.21	0.23	-0.54	-0.37
8 9	0.55	0.72	0.01	0.04	0.42	0.90	0.23	0.12	-0.59	0.27	-0.31	-0.58	-0.17	-0.05	-0.86	-0.23
9 10	0.13	-0.11	0.23	-0.55	0.65	1.04	0.09	-0.06	-0.64	0.01	0.37	-0.23	0.27	-0.02	-1.32	-0.41
10 11	0.49	0.33	-0.49	-0.02	1.04	1.09	-0.37	0.06	0.45	0.50	-0.01	-0.40	-0.28	0.24	-1.14	-0.52
11 12	0.17	0.80	0.30	0.04	0.56	1.02	0.04	0.62	-0.76	0.51	-0.33	-0.60	-0.34	0.09	-0.56	-0.41
12 13	-0.25	-0.14	-0.22	-0.26	1.30	0.89	-0.01	0.46	-0.51	0.09	0.36	-0.24	-0.10	0.14	-0.76	-0.24
13 14	0.25	0.34	-0.40	-0.46	0.53	0.98	-0.20	0.06	-0.03	0.28	0.17	-0.48	0.16	0.05	-1.11	-0.02
14 15	0.23	0.50	-0.03	0.20	0.70	1.07	-0.06	0.18	-0.40	0.16	-0.09	-0.59	-0.19	0.30	-1.52	-0.23
15 16	-0.15	0.24	0.12	-0.36	0.86	0.96	0.30	0.19	-0.26	-0.01	-0.32	-0.27	-0.11	-0.03	-0.77	-0.07
16 17	0.31	0.35	-0.38	-0.27	0.81	1.30	-0.61	0.21	0.11	0.23	-0.15	-0.34	-0.21	0.26	-1.03	-0.10
17 18	0.20	0.49	0.16	-0.28	1.05	1.29	-0.11	0.45	-0.56	-0.08	-0.37	-0.64	-0.17	0.09	-0.82	-0.06
18 19	0.21	-0.13	-0.12	-0.15	0.97	0.88	0.08	0.04	-0.50	0.32	0.00	-0.39	-0.05	0.09	-0.91	-0.16
19 20	0.61	0.24	-0.20	-0.47	0.89	0.80	-0.10	0.05	0.10	0.43	-0.42	-0.32	-0.17	0.07	-0.59	-0.26
20 21	0.84	0.43	0.03	-0.25	0.93	1.06	-0.09	-0.01	-0.35	0.44	-0.32	-0.58	-0.43	0.06	-1.13	-0.09
21 22	-0.05	0.41	0.13	-0.17	0.93	0.79	0.16	0.21	-0.43	0.19	-0.16	-0.44	0.04	0.01	-1.05	-0.49
22 23	0.31	0.21	0.06	-0.67	0.94	1.01	-0.09	0.04	-0.03	0.45	-0.12	-0.43	-0.20	0.10	-1.21	-0.36
23 24	0.48	0.41	-0.05	-0.18	0.54	0.70	0.02	0.63	-0.31	0.37	-0.09	-0.48	-0.10	-0.27	-0.90	-0.41
24 25	0.15	0.17	-0.01	0.10	0.54	0.55	0.12	-0.12	-0.43	0.29	-0.08	-0.17	0.15	-0.20	-0.59	-0.25
25 26	0.52	0.31	-0.15	-0.38	0.70	0.57	-0.07	-0.11	-0.01	0.19	-0.03	-0.26	0.10	-0.02	-0.85	-0.19
26 27	0.58	0.42	0.27	-0.30	0.52	0.63	0.01	-0.02	-0.43	0.35	-0.15	-0.31	-0.17	0.00	-0.75	-0.37
27 28	-0.04	0.21	0.00	-0.05	1.00	0.71	0.01	-0.02	-0.24	0.30	0.32	-0.42	-0.31	-0.04	-0.74	-0.28
28 29	0.18	0.68	-0.50	-0.20	0.61	0.87	0.00	0.02	-0.01	0.64	-0.20	-0.10	-0.07	-0.19	-1.11	-0.09
29 30	0.38	-0.03	0.04	-0.02	0.72	1.01	-0.17	0.33	-0.50	-0.01	-0.25	-0.45	-0.09	0.19	-0.81	-0.06
30 31	0.25	0.04	-0.06	-0.12	0.74	0.57	0.10	0.05	-0.23	0.22	-0.11	-0.64	0.13	-0.01	-0.09	-0.31
31 32	0.32	0.44	-0.05	-0.03	0.72	0.58	-0.15	0.04	0.05	-0.05	0.12	-0.24	-0.29	-0.17	-1.05	-0.16
32 33	0.42	0.29	0.09	-0.47	0.31	0.94	-0.46	0.41	-0.06	-0.09	-0.02	-0.32	-0.17	0.28	-0.99	-0.14
33 34	0.39	0.19	0.11	-0.48	0.88	1.04	0.03	-0.14	-0.33	0.20	-0.24	-0.46	-0.06	0.02	-0.74	-0.14
34 35	0.45	0.65	-0.20	-0.25	0.79	1.01	-0.04	-0.06	-0.44	0.50	0.21	-0.57	-0.31	0.10	-0.22	-0.64
35 36	0.36	0.07	-0.23	-0.22	0.82	1.02	0.28	0.28	0.18	-0.10	0.00	-0.24	-0.50	-0.22	-1.01	-0.26
36 37	0.34	0.19	0.02	-0.21	0.66	0.89	-0.02	-0.52	-0.43	0.16	0.15	-0.12	0.06	-0.01	-1.09	-0.11
37 38	0.39	0.34	-0.21	-0.11	1.23	0.97	-0.18	-0.27	-0.19	0.19	-0.02	-0.20	-0.36	0.29	-1.25	-0.30
38 39	0.09	0.29	0.26	-0.50	0.80	1.14	0.21	-0.10	-0.53	0.00	-0.05	-0.36	-0.41	0.05	-0.37	-0.29
39 40	-0.03	0.25	-0.23	-0.42	0.91	0.69	0.09	0.18	-0.19	0.26	0.45	-0.14	-0.23	-0.10	-0.87	-0.35
40 41	-0.03	0.44	-0.19	-0.20	0.43	0.52	-0.04	0.24	-0.39	0.17	0.33	-0.15	-0.03	-0.08	-0.56	-0.26
41 42	-0.19	0.49	-0.10	-0.33	0.75	0.79	-0.23	-0.01	-0.66	0.48	0.22	0.01	-0.26	0.16	-1.25	0.16
42 43	-0.20	-0.26	-0.13	-0.34	0.86	0.41	0.39	0.34	0.34	-0.07	-0.41	-0.33	0.00	0.15	-0.29	-0.11

43 44	0.49	0.48	0.06	-0.34	0.74	0.45	-0.29	-0.10	-0.20	-0.08	0.22	-0.35	-0.31	0.06	-0.80	0.12
44 45	-0.15	0.29	0.21	-0.17	0.58	0.99	-0.29	0.03	-0.26	-0.50	0.31	-0.19	-0.23	0.32	-0.98	-0.23
45 46	-0.32	0.08	-0.08	-0.08	0.61	0.46	-0.14	0.52	-0.16	-0.20	0.25	-0.04	-0.08	-0.07	-0.49	-0.19
46 47	-0.27	0.50	-0.31	-0.06	0.16	0.98	-0.25	-0.38	-0.35	0.11	0.03	0.11	-0.38	0.39	-0.45	0.21
47 48	-0.36	0.50	0.15	-1.12	0.03	1.72	0.69	-0.20	-0.90	1.54	0.63	-0.77	-0.44	1.20	-0.21	-0.25
48 49	-1.36	0.38	0.09	0.40	0.99	1.60	1.10	1.17	-0.56	0.30	1.31	1.29	-1.65	0.44	-0.32	1.00
49 50	1.17	1.67	-2.42	0.78	3.13	2.91	-0.56	1.86	1.76	2.40	-0.14	1.16	1.82	2.10	-1.86	1.82
50 51	0.00	0.00	1.72	0.00	0.00	0.00	2.18	0.00	0.00	0.00	-1.48	0.00	0.00	0.00	1.41	0.00
51 52	0.00	0.00	0.00	0.00	0.00	0.00	0.00	0.00	0.00	0.00	0.00	0.00	0.00	0.00	0.00	0.00
52 53	0.00	0.00	0.00	0.00	1.48	3.25	-0.41	2.78	0.00	0.00	0.00	0.00	0.00	0.00	0.00	0.00
53 54	-2.56	-0.68	-0.52	-0.60	1.73	4.17	2.71	4.74	-2.08	2.59	1.03	1.45	1.42	3.67	2.42	5.21
54 55	0.98	0.78	-3.25	1.58	2.42	3.85	0.39	3.71	3.27	3.08	-0.82	3.05	2.15	2.50	-0.57	1.69
55 56	2.38	1.79	1.55	2.15	2.02	2.12	2.03	3.11	-1.28	-2.88	-1.24	-1.23	2.32	2.18	2.14	2.54
56 57	0.62	0.96	0.26	1.01	1.41	0.30	-1.20	-0.15	-0.20	0.95	0.18	-0.26	0.34	0.94	-0.68	0.68
57 58	0.09	-0.30	-0.29	-0.36	1.10	0.92	0.36	0.73	-0.30	-0.12	-0.58	-0.78	0.53	0.52	0.08	-0.18
58 59	0.41	0.46	0.18	-0.06	0.70	0.53	0.27	-0.20	0.05	0.55	-0.50	-0.51	0.20	-0.20	-0.47	-0.53
59 60	0.73	0.32	0.14	0.02	0.83	0.47	-0.08	0.23	0.87	0.55	-0.69	-0.24	-0.21	-0.04	-0.87	-0.65
60 61	0.46	1.11	0.05	0.32	0.26	0.39	0.14	0.33	-0.09	0.37	-0.73	-0.35	-0.09	0.06	-0.42	-0.60
61 62	0.38	0.49	-0.22	-0.09	0.69	0.38	0.29	0.39	0.20	0.53	-0.84	-0.47	0.12	0.07	-0.26	-0.54
62 63	0.35	0.63	0.19	0.13	0.78	0.02	0.29	0.44	-0.12	-0.22	-0.56	-0.16	0.37	-0.13	-0.52	-0.78
63 64	0.82	0.23	0.02	0.35	0.97	0.25	0.01	-0.12	0.27	0.25	-0.63	0.07	0.12	0.15	-0.46	-0.53
64 65	0.72	0.76	0.18	0.25	0.08	0.28	-0.07	0.13	0.13	-0.18	-0.55	-0.31	0.35	-0.01	-0.50	-0.48
65 66	0.62	0.56	-0.03	0.26	0.60	0.13	-0.11	0.37	0.21	0.27	-0.81	-0.08	0.12	-0.06	-0.42	-0.43
66 67	0.82	0.11	0.09	0.42	0.52	0.18	-0.11	0.31	0.07	0.08	-0.96	-0.03	0.40	0.17	-0.33	-0.48
67 68	0.71	0.48	0.16	0.61	0.11	0.14	0.12	0.20	-0.18	0.00	-0.82	-0.27	0.29	0.05	-0.46	-0.27
68 69	0.49	0.17	0.08	0.18	0.35	0.12	-0.08	0.39	0.08	-0.02	-0.79	0.02	0.36	0.22	-0.45	-0.37
69 70	0.34	0.70	-0.07	0.51	0.54	0.37	-0.28	0.17	-0.20	0.06	-0.61	-0.35	0.29	0.01	-0.19	-0.20
70 71	0.47	0.30	-0.14	0.37	0.72	0.49	0.07	-0.08	0.06	-0.15	-0.89	-0.01	-0.11	0.46	-0.50	-0.35
71 72	0.64	0.21	-0.07	0.16	0.44	0.23	0.26	0.36	-0.04	0.02	-0.86	-0.18	0.26	0.09	-0.35	-0.40
72 73	0.56	0.29	0.08	0.36	0.42	0.12	0.18	0.19	0.13	0.31	-1.03	-0.10	0.39	0.01	-0.37	-0.37
73 74	0.57	0.56	0.17	0.27	0.34	0.16	-0.04	0.02	-0.06	-0.17	-0.72	-0.06	0.16	-0.11	-0.42	-0.04
74 75	0.58	0.27	0.02	0.15	0.81	0.28	-0.08	-0.25	0.04	0.19	-0.77	-0.13	0.14	0.22	0.05	-0.25
75 76	0.34	0.72	-0.13	0.67	0.76	0.43	0.05	0.00	0.08	-0.12	-0.81	0.04	-0.12	-0.08	-0.18	-0.25
76 77	0.51	0.15	-0.07	0.28	0.53	0.39	-0.02	0.11	0.13	0.28	-1.16	-0.13	0.29	0.18	-0.12	-0.27
77 78	0.82	0.53	0.06	0.04	0.18	0.50	0.08	0.23	-0.05	-0.31	-0.78	-0.37	0.03	0.11	0.00	-0.25
78 79	0.56	0.42	-0.22	0.27	0.72	0.35	-0.08	0.07	-0.18	0.25	-0.67	-0.03	0.17	-0.28	-0.22	-0.23
79 80	0.60	0.18	0.06	0.24	0.06	0.18	0.19	0.11	-0.04	-0.08	-0.87	0.00	0.27	0.05	0.20	-0.40
80 81	0.30	0.58	-0.04	0.33	0.43	0.41	0.13	-0.52	-0.10	0.07	-0.75	0.33	0.02	0.21	-0.45	-0.25
81 82	0.16	0.28	-0.02	0.16	0.31	0.38	0.26	0.28	-0.18	0.32	-0.60	-0.30	-0.06	0.15	-0.63	-0.13
82 83	0.32	0.57	-0.38	-0.32	0.30	0.10	0.34	0.38	0.09	0.22	-0.71	-0.15	0.10	0.13	-0.09	-0.26
83 84	0.52	0.24	-0.13	0.30	0.30	0.13	0.16	0.35	-0.45	0.23	-0.51	-0.17	-0.19	0.09	0.09	-0.24
84 85	0.40	0.33	-0.33	-0.27	0.50	0.48	-0.15	0.02	-0.04	0.08	-0.31	-0.15	0.20	-0.16	-0.13	0.05
85 86	0.42	0.34	0.14	0.08	0.53	0.18	-0.07	0.18	0.28	-0.17	-1.05	0.40	0.02	-0.07	-0.09	-0.11
86 87	0.51	0.50	0.14	-0.04	0.47	0.19	-0.18	-0.05	-0.40	-0.09	-0.73	-0.04	0.12	0.24	-0.10	0.09
87 88	0.37	0.31	-0.28	0.26	0.25	0.42	0.05	0.18	-0.08	0.35	-0.84	-0.10	0.07	0.23	-0.16	-0.26
88 89	0.12	0.57	-0.24	0.37	0.76	0.36	0.08	0.26	-0.27	-0.19	-0.62	-0.22	0.08	0.06	-0.42	-0.08
89 90	0.20	0.37	-0.18	0.33	0.26	0.39	-0.10	0.29	-0.12	-0.15	-0.50	-0.26	0.20	-0.07	0.17	0.02
90 91	0.25	0.34	-0.23	0.30	0.35	0.40	0.03	-0.24	-0.34	-0.06	-0.38	-0.09	0.41	0.29	-0.44	-0.30
91 92	0.28	0.36	-0.06	0.10	0.27	0.62	0.12	0.05	-0.06	0.06	-0.58	-0.08	-0.13	0.07	0.11	-0.35
92 93	0.24	0.34	-0.45	0.54	0.32	0.10	0.21	0.51	0.08	0.12	-0.66	0.04	-0.06	0.18	0.09	-0.50
93 94	0.22	0.51	-0.36	0.41	0.42	0.28	-0.04	0.21	-0.20	0.00	-0.57	-0.18	0.37	-0.17	-0.21	0.06
94 95	0.41	0.71	-0.05	-0.26	0.43	0.25	-0.16	0.13	-0.37	-0.11	-0.54	-0.17	0.20	0.23	0.05	-0.15
95 96	0.25	0.29	-0.19	0.37	0.29	0.22	0.18	0.36	-0.30	0.30	-0.78	0.12	0.23	-0.02	-0.05	-0.47
96 97	0.03	0.35	0.02	0.03	0.29	0.25	0.08	0.18	-0.09	0.26	-0.52	-0.42	0.18	0.34	-0.64	-0.25
97 98	0.38	0.00	0.03	-0.09	0.35	0.17	0.22	0.52	-0.03	0.19	-0.61	-0.09	-0.07	-0.10	-0.42	-0.18
98 99	0.57	0.38	-0.18	-0.19	0.20	0.10	-0.09	0.12	0.19	-0.01	-0.48	-0.22	0.08	0.13	0.07	-0.11
99 100	0.35	0.65	-0.18	0.40	0.19	0.21	-0.06	0.29	-0.22	0.28	-0.60	-0.17	-0.04	-0.07	-0.36	-0.11
100 101	0.61	-0.16	-0.08	-0.13	0.12	0.28	0.30	0.16	-0.28	-0.03	-0.62	-0.14	0.10	0.26	-0.11	-0.10
101 102	0.29	0.12	-0.11	0.20	0.40	0.27	-0.03	-0.03	-0.25	0.53	-0.55	-0.09	0.79	-0.14	-0.33	-0.52

Using the above difference matrix the following sequence is encoded into a numeric vector of 101 observations

>Sequence
TCCAGACCTTCTGCCAGAAAGGGGGCCTGTTGTGCACGCTTCAGGGCAAGGTGGGGCTGCCTGCCTGCCT
GAGGGCTGAAGGGCACAGGGTCTGTGGGAGGG

Di-nucleotide combinations at adjacent positions for above sequence

1_2	2_3	3_4	…	…	…	99_100	100_101	101_102
TC	CC	CA	…	…	…	AG	GG	GG

Based on the above combination of di-nucleotides and adjacent positions, the sequence is encoded in to numeric vector (0.35, -0.61, 0.03, -0.08, -0.40, 0.02, -0.37, -0.05, 1.04, 0.06, 0.09, -0.01, -0.48, -0.23, -0.11, -0.38, -0.56, 0.21, 0.61, 0.03, -0.16, -0.12, -0.09, -0.08, -0.26, -0.37, -0.09, 0.00, -0.01, 0.57, -0.15, -0.09, 0.03, -0.57, -0.50, -0.21, 0.29, -0.36, -0.10, 0.52, 0.01, 0.00, 0.06, 0.31, 0.25, 0.11, -0.44, -1.36, -2.42, -1.48, 0.00, -0.41, 1.03, -0.82, -1.24, -0.26, 0.52, 0.27, 0.24, -0.60, 0.07, 0.29, 0.07, -0.48, -0.06, -0.11, -0.27, -0.37, 0.01, 0.07, -0.04, 0.08, -0.72, -0.77, 0.44, 0.18, 0.08, -0.18, 0.60, -0.04, -0.60, -0.71, -0.17, 0.20, 0.08, 0.12, -0.28, -0.62, -0.50, -0.06, 0.05, 0.18, -0.04, -0.11, 0.18, -0.52, -0.61, 0.19, -0.18, -0.62, -0.55), where each observation of the vector is taken from the difference table (marked with red color). In this way, each true, false and test sequence was encoded into numeric vector. In true and false splice sites, labels were retained in the encoded vector and were together used as input to train the model. Then, the label (probability) of encoded test sequence is computed using the trained model.
