# Supplementary material for: Prediction of donor splice sites using random forest with a new sequence encoding approach
Source: BioData Min. 2016 Jan 22;9:4. doi: 10.1186/s13040-016-0086-4 (PMC4724119; doi:10.1186/s13040-016-0086-4)
Supplement: Supplementary file 2 — Plotting of margin function for encoding procedure 2 (P-2). Description of the data: Each dot in the plot is the value of margin function for an observation (TSS or FSS). Ten different plots corresponding to 10 test sets of the 10-fold cross validation. Red and blue points are the values of margin function for FSS and TSS. The values above zero indicate that the instances are correctly classified. (PDF 110 kb) [file 13040_2016_86_MOESM2_ESM.pdf]

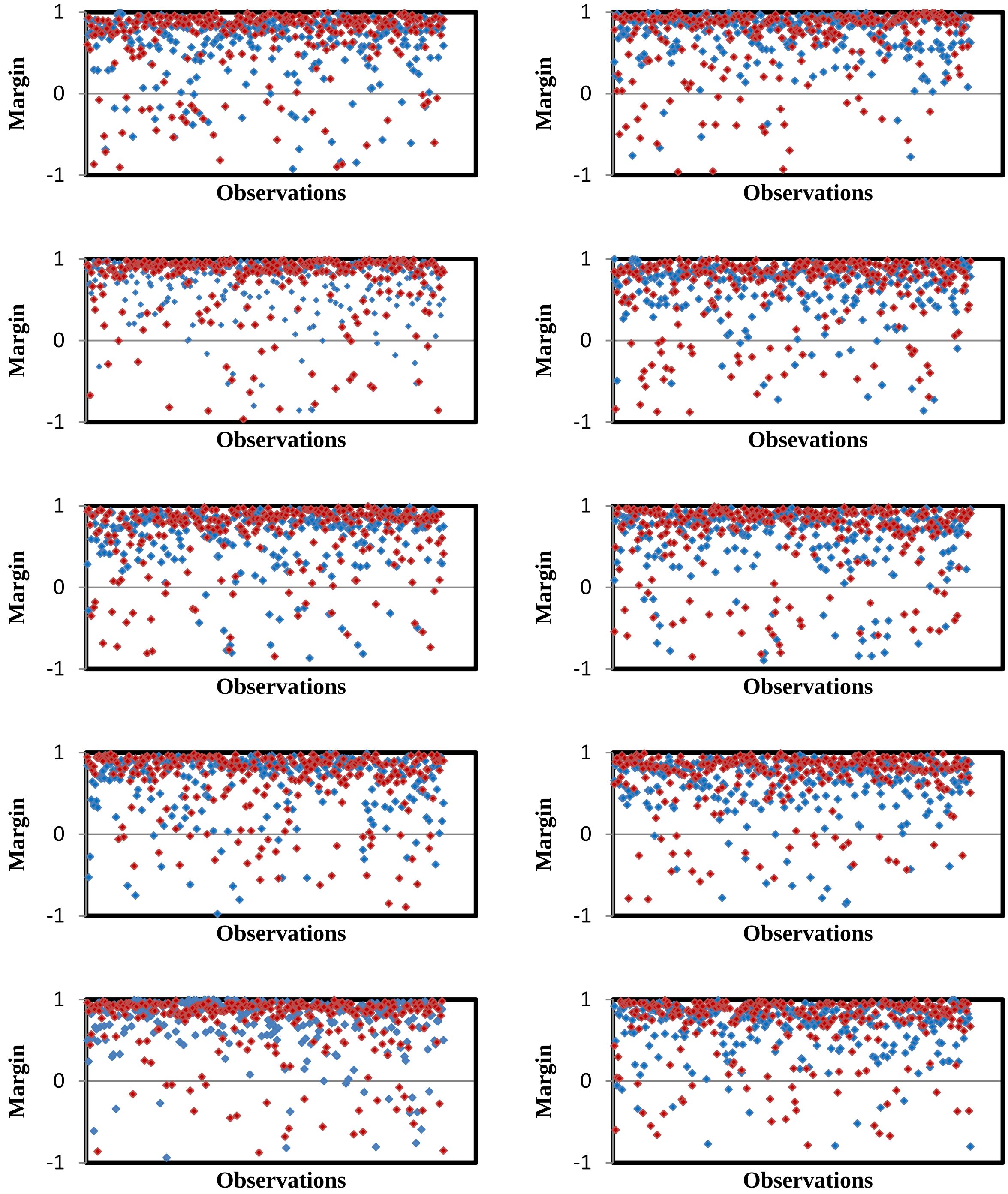

Plotting of margin function for P-2
